# The frequency of ANCA-associated vasculitis in a national database of hospitalized patients in China

**DOI:** 10.1186/s13075-018-1708-7

**Published:** 2018-10-04

**Authors:** Jiannan Li, Zhao Cui, Jian-yan Long, Wei Huang, Jin-wei Wang, Haibo Wang, Luxia Zhang, Min Chen, Ming-hui Zhao

**Affiliations:** 1 Renal Division, Department of Medicine, Peking University First Hospital, Peking University Institute of Nephrology, Key Laboratory of Renal Disease, Ministry of Health of China, Key Laboratory of CKD Prevention and Treatment, Ministry of Education of China, Beijing, China; 2 Clinical Trial Unit, First Affiliated Hospital of Sun Yat-Sen University, Guangzhou, China; 3 Department of Occupational and Enviromental Health, Peking University School of Public Health, Beijing, China; 4 China Standard Medical Information Research Center, Shenzhen, Guangdong China; 5 Peking University, Center for Data Science in Health and Medicine, Beijing, China; 6 Peking-Tsinghua Center for Life Sciences, Beijing, People’s Republic of China

**Keywords:** ANCA, Vasculitis, Frequency, Hospitalized population

## Abstract

**Background:**

Anti-neutrophil cytoplasmic autoantibody (ANCA)-associated vasculitis (AAV) is a group of life-threatening autoimmune diseases. The epidemiological data on AAV in China are limited. The aim of the present study is to investigate the frequency, geographical distribution, and ethnic distribution of AAV in hospitalized patients in China, and its association with environmental pollution.

**Methods:**

We investigated the hospitalized patients in a national inpatient database covering 54.1% tertiary hospitals in China from 2010 to 2015. Diagnosis of AAV was extracted according to the definition of International Classification of Diseases (ICD)-10 codes and free text. Variables from the front page of inpatient records were collected and analyzed, including frequency, geographic distribution, demographic characteristics and seasonal variations of AAV. The association between various environmental pollutants and frequency of AAV was further analyzed.

**Results:**

Among 43.7 million inpatients included in the study period, 0.25‰ (10,943) were diagnosed as having AAV. The frequency of AAV was relatively stable during the study period (from 0.34‰ in 2010 to 0.27‰ in 2015). The proportion of AAV increased with latitude (0.44‰ in Northern China and 0.27‰ in Southern China in 2015). Hospitalizations were mostly observed in winter (30.2%). The Dong population, an ethnic minority of the Chinese population, had the highest frequency of patients with AAV (0.67‰). We also found a positive association between the exposure to carbon monoxide and the frequency of AAV (*R*^2^ = 0.172, *p* = 0.025). In Yunnan province, the frequency of AAV increased 1.37-fold after the Zhaotong earthquake, which took place in 2014.

**Conclusions:**

Our present investigation of hospitalized patients provided epidemiological information on AAV in China for the first time. A spatial and ethnic clustering trend and an association between pollution and the frequency of AAV were observed.

**Electronic supplementary material:**

The online version of this article (10.1186/s13075-018-1708-7) contains supplementary material, which is available to authorized users.

## Background

Anti-neutrophil cytoplasmic autoantibody (ANCA) associated vasculitis (AAV) is a group of life-threatening autoimmune diseases affecting mainly small-to-medium vessels [1], with a poor prognosis if left untreated [2–8]. According to the 2012 classification of the Chapel Hill Consensus Conference (CHCC) [9], the phenotypes include four clinical syndromes, namely, granulomatosis with polyangiitis (GPA, formerly known as Wegener’s granulomatosis), microscopic polyangiitis (MPA), eosinophilic granulomatosis with polyangiitis (EGPA, formerly known as Churg-Strauss syndrome), and single-organ AAV (for example, renal-limited AAV). The serological marker for AAV is ANCA [10].

The epidemiological characteristics of AAV have been investigated worldwide. The annual incidence and prevalence of AAV varies according to latitude in both the southern and northern hemispheres [11]. Several environmental factors are associated with the development of AAV. Exposure to silicons and subsequent modification of myeloperoxidase (MPO) by air pollution is suspected to be a risk factor for AAV [12–14]. Earthquake might also be a source of silicons, which might subsequently result in the incremental incidence of AAV with more rapid deteriotion of renal function [15, 16]. However, this phenomenon was not supported by a similar study in New Zealand [17]. Infections might contribute to the onset of AAV, especially *Staphylococcus aureus* infections [18–20]. Moreover, certain genetic backgrounds might lead to greater susceptiblity to AAV [21], especially in specific races [22]. However, although AAV was first reported in 1993 in China [23, 24], only limited single-center surveys of AAV have been carried out [25, 26] and nationwide epidemiological investigations are not yet available. The purpose of the present study was to investigate the proportion and characteristics of AAV patients and their clinical phenotypes in hospitalized patients in China.

## Methods

### Study population

The study population included 43,677,829 inpatients from 878 tertiary hospitals from 1 Jan 2010 to 31 Dec 2015, covering 54.1% of tertiary hospitals in 31 provinces nationwide.

The database we used is the Hospital Quality Monitoring System (HQMS), which is a registration database of the standardized electronic inpatient discharge records of tertiary hospitals in China. Under the administration of the Bureau of Medical Administration and Medical Service Supervision, National Health and Family Planning Commission of the People’s Republic of China, tertiary hospitals in China have mandatorily and automatically submitted electronic discharge records daily to HQMS, since 1 Jan 2013. Data from 1 Jan 2010 to 31 Dec 2012 were collected retrospectively. Demographic characteristics, clinical diagnoses, procedures, pathological diagnoses, and expenditures were extracted from the front page of the hospital medical record.

Physicians were responsible for filing the data on the front page, and the diagnosis were coded by certified professional medical coders at every hospital according to the International Classification of Diseases-10 (ICD-10) coding system. Data quality was controlled automatically at the time of data submission to ensure completeness, consistency, and accuracy.

For patients with multiple admissions, only the first admission was included for analysis. We identified 288,804 patients for analysis from 1 Jan 2013 to 31 Dec 2015, and 11,102 patients from 1 Jan 2010 to 31 Dec 2012. Identification numbers and telephone numbers were combined to define the place of patient residence. Urban/rural residency was identified by the type of health insurance (basic medical insurance or free medical insurance for urban residency, and new rural cooperative medical care for rural residency). The ethics committee of Peking University First Hospital approved the study.

### Definition of AAV

The ICD-10 coding of discharged diagnoses and free text were used to identify patients with AAV compromising granulomatosis with polyangiitis (GPA), microscopic polyangitis (MPA), eosinophilic granulomatosis with polyangiitis (EGPA) and kidney-limited vasculitis (relevant ICD-10 coding in Appendix 1). The definition of AAV had to exclude large vessel vasculitis (e.g., Takayasu arteritis, giant cell arteritis), medium vessel vasculitis (e.g., polyarteritis nodosa, Kawasaki disease), and immune complex small vessel vasculitis (SVV) (e.g., rheumatoid vasculitis, sarcoid vasculitis, and others) (relevant ICD-10 coding in Appendix 2), from which, 6844 patients were excluded. Nephrotic syndrome, rapidly progressive glomerulonephritis, nephritis syndrome, and related complications are also listed in Appendix 2.

### Demographic data and other covariates

Information on age, gender, ethnicity, occupation, residence, health insurance, type of admission, and intensive care unit (ICU) stay were collected from the front page of the medical records. Outcome data on expenditure, length of stay, and in-hospital mortality were also extracted. The survival status of each patient was verified based on discharge status, and combined with information from autopsy reporting.

### Geographic latitude

The latitude and longitude of each province and each capital city in China were acquired from the National Bureau of Statistics (http://www.stats.gov.cn/). The range of latitude of Northeastern China is 38.7° N to 53.6° N, of Northern China it is 34.9° N to 53.4° N, of Northwestern China it is 31.7° N to 48.2° N, of Central China it is 24.6° N to 36.4° N, of Eastern China it is 23.5° N to 38.4° N, of Southern China it is 18.2° N to 26. 4° N, and of Southwestern China it is 20° N to 34.3° N.

### Pollution exposure assessment

The National Bureau of Statistics of China has published the average concentrations of air pollutants in each city, including main pollutant emission in waste gas, which contained particulate matter (PM) of 2.5 (μg/m^3^) (PM 2.5) in 2015, PM of 10 (μg/m^3^) (PM 10) since 2010, carbon monoxide (CO) (μg/m^3^), inhalable particulate (10,000 tons), nitrogen dioxide (NO_2_) (10,000 tons), sulfur oxide (SO_2_) (10,000 tons) since 2002; main pollutant emission in waste water, which contains the total volume of waste water discharged (10,000 tons), ammonia nitrogen (10,000 tons), total nitrogen (10,000 tons), total phosphorus (10,000 tons), petroleum (ton), volatile phenol (ton), plumbum (kg), mercury (kg); and general industrial solid waste per year since 2002, which contains household garbage and industrial solid wastes, with various kinds of pollutants, including nitrogenous wastes, organic pollutants, such as polycyclic aromatic hydrocarbons, which mainly affects soil and water. Data on polycyclic aromatic hydrocarbon pollution in soil has been provided by Ma et al. [27] Data on seasonal PM 2.5 and PM 10 in 2014–2015 has been provided by Zhang et al. [28] Data on annual average temperature, humidity, and precipitation were acquired from the National Meteorological Center (http://data.cma.cn). Detailed data on pollutants are listed in Additional files 1, 2, and 3. 

### Statistical analyses

The proportion and absolute number of patients with AAV were identified and further analyzed. Patients with AAV were stratified by age, gender, geographic regions, and rural/urban residency. General demographic characteristics, costs, length of stay and in-hospital mortality were compared among patients with GPA, MPA, and EGPA. Continuous data were analyzed as mean ± standard deviation, or as median (inter-quartile range) for highly skewed variables. Categorical variables were analyzed as proportions with 95% confidence interval (CI).

The association between the frequency of AAV in the hospitalized population and exposure to ambient environmental pollution were analyzed using Pearson correlation models and generalized linear regression models. Data on air pollutants were adjusted for annual average temperature, humidity, and precipitation. The proportions of patients with AAV were adjusted for average age and gender. The analysis of populations of patients with AAV was based on individual patients instead of admissions, since the number of patients was more relevant to the prevalence of AAV. All analyses were performed using SAS software, version 9.1 (SAS Institute Inc., Cary, NC, USA).

## Results

### Demographic characteristics of patients with AAV in 2015

**Fig. 1:**
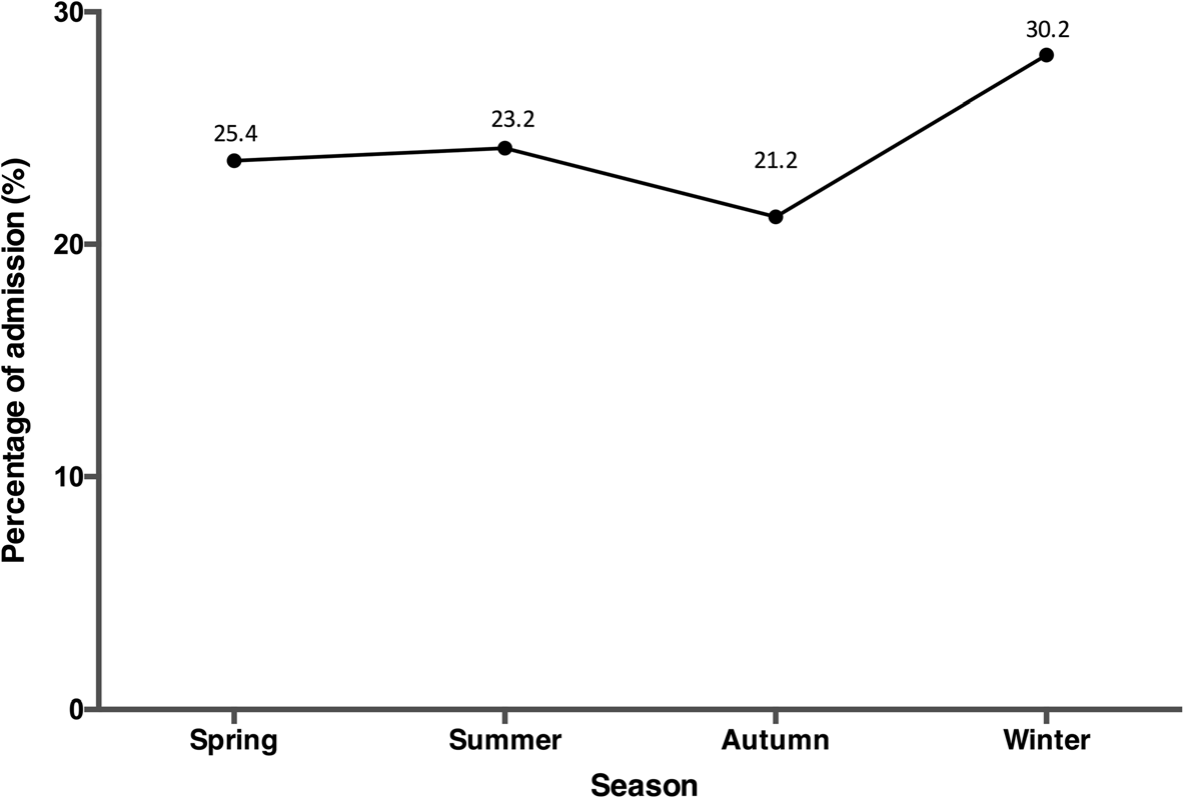
The distribution of anti-neutrophil cytoplasmic autoantibody associated vasculitis (AAV) in different seasons in 2015. The highest frequency of admissions for AAV were in winter (30.2%)

**Fig. 2:**
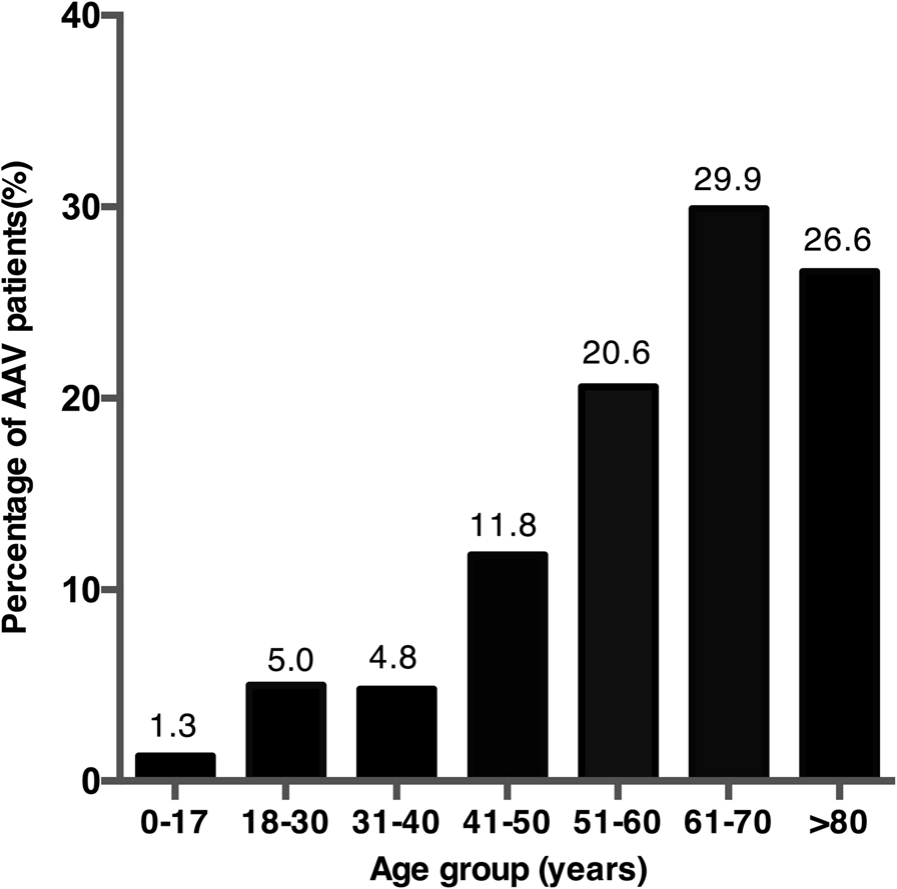
The age distribution of anti-neutrophil cytoplasmic autoantibody associated vasculitis (AAV) in 2015. Most patients with AAV were older than 50 years, with a peak age at 61–70 years (29.9%)

The frequency of AAV was relatively stable in the study period (from 0.34‰ in 2010 to 0.27‰ in 2015). There were 4440 patients (0.27‰ of all inpatients) identified as having AAV in 2015 and these were included for further analysis. The demographic characteristics of patients with AAV in 2015 are shown in Table 1. Patients with AAV were most commonly admitted by the nephrology division (*n* = 1971 (44.4%)), followed by the respiratory division (*n* = 584 (13.2%)), and the rheumatology division (*n* = 509 (11.5%)). Most admissions were in winter (30.2%) (Fig. 1). The age of patients with AAV at diagnosis was 60.0 ± 15.6 years, and the majority were older than 50 years (Fig. 2).

**Table 1:** Demographic information on patients with AAV in 2015 in China

	AAV	GPA	MPA	EGPA
Number	4440	385	396	223
Age (years)	60.0 ± 15.6	50.7 ± 15.6	62.3 ± 17.2	50.3 ± 15.6
Age group, %				
0–17	1.3	1.6	3.5	3.1
18–30	5.0	11.4	2.8	9.4
31–40	4.8	10.4	2.8	11.2
41–50	11.8	23.9	7.4	20.2
51–60	20.6	24.4	17.4	27.8
61–70	29.9	17.1	34.8	21.5
> 80	26.6	11.2	31.3	6.6
Male, %	46.4(45.0,47.9)	49.9(44.9,54.9)	48.5(43.6,53.4)	52.5(45.9,59.0)
Occupation, %				
Professional or semi-professional	9.1 (8.2,10.0)	14.7 (10.9,18.4)	8.5 (5.6,11.4)	16.1 (11.1,21.1)
Worker	2.9 (2.4,3.4)	2.9 (1.1,4.6)	2.5 (0.9,4.2)	3.9 (1.3,6.6)
Farmer	25.7 (24.3,27.0)	22.4 (18.0,26.8)	25.4 (20.9,30.0)	22.0 (16.3,27.6)
Retired	20.4 (19.1,21.6)	12.9 (9.4,16.5)	24.3 (19.8,28.8)	15.6 (10.6,20.6)
Unemployed	7.2 (6.4,8.0)	6.9 (4.2,9.6)	7.9 (5.1,10.7)	4.4 (1.6,7.2)
Others	34.7 (33.3,36.2)	40.2 (35.1,45.4)	31.4 (26.5,36.2)	38.0 (31.4,44.7)
Medical insurance				
Basic Medical Insurance	44.4 (43.0,45.9)	38.2 (33.3,43.0)	44.4 (39.6,49.3)	45.7 (39.2,52.3)
New Rural Co-operative Medical Care	24.1 (22.9,25.4)	24.7 (20.4,29.0)	21.7 (17.7,25.8)	25.1 (19.4,30.8)
Other insurance	16.0 (14.9,17.1)	16.1 (12.4,19.8)	21.5 (17.4,25.5)	17.9 (12.9,23.0)
No insurance	15.4 (14.4,16.5)	21.0 (17.0,25.1)	12.4 (9.1,15.6)	11.2 (7.1,15.4)
Admission place				
Emergency	12.6 (11.6,13.6)	15.0 (11.3,18.7)	11.5 (8.2,14.7)	16.4 (11.5,21.4)
Routine	79.9 (78.7,81.1)	76.7 (72.3,81.0)	83.1 (79.2,86.9)	77 (71.3,82.6)
Other	7.5 (6.7,8.3)	8.3 (5.5,11.2)	5.5 (3.1,7.8)	6.6 (3.2,9.9)
ICU stay, %	2.2 (1.7,2.6)	0.3 (0,0.8)	3.5 (1.7,5.4)	0.4 (0,1.3)
Costs (10,000 RMB), median (Q1–Q3)	13 (7–23)	10 (6–19)	14 (8–24)	11 (7–18)
Length of stay (days), median (Q1–Q3)	12 (8–19)	12 (7–18)	13 (8–21)	12 (8–16)
In-hospital mortality, %	1.8 (1.4,2.2)	1.3 (0.2,2.4)	2.8 (1.2,4.4)	0.4 (0,1.3)

*Abbreviations:* *AAV* anti-neutrophil cytoplasmic autoantibody associated vasculitis, *GPA* granulomatosis with polyangiitis, *MPA* microscopic polyangiitis, *EGPA* eosinophilic granulomatosis with polyangiitis, *ICU* intensive care unit, *RMB* Renminbi, which is Chinese currency.

**Fig. 3:**
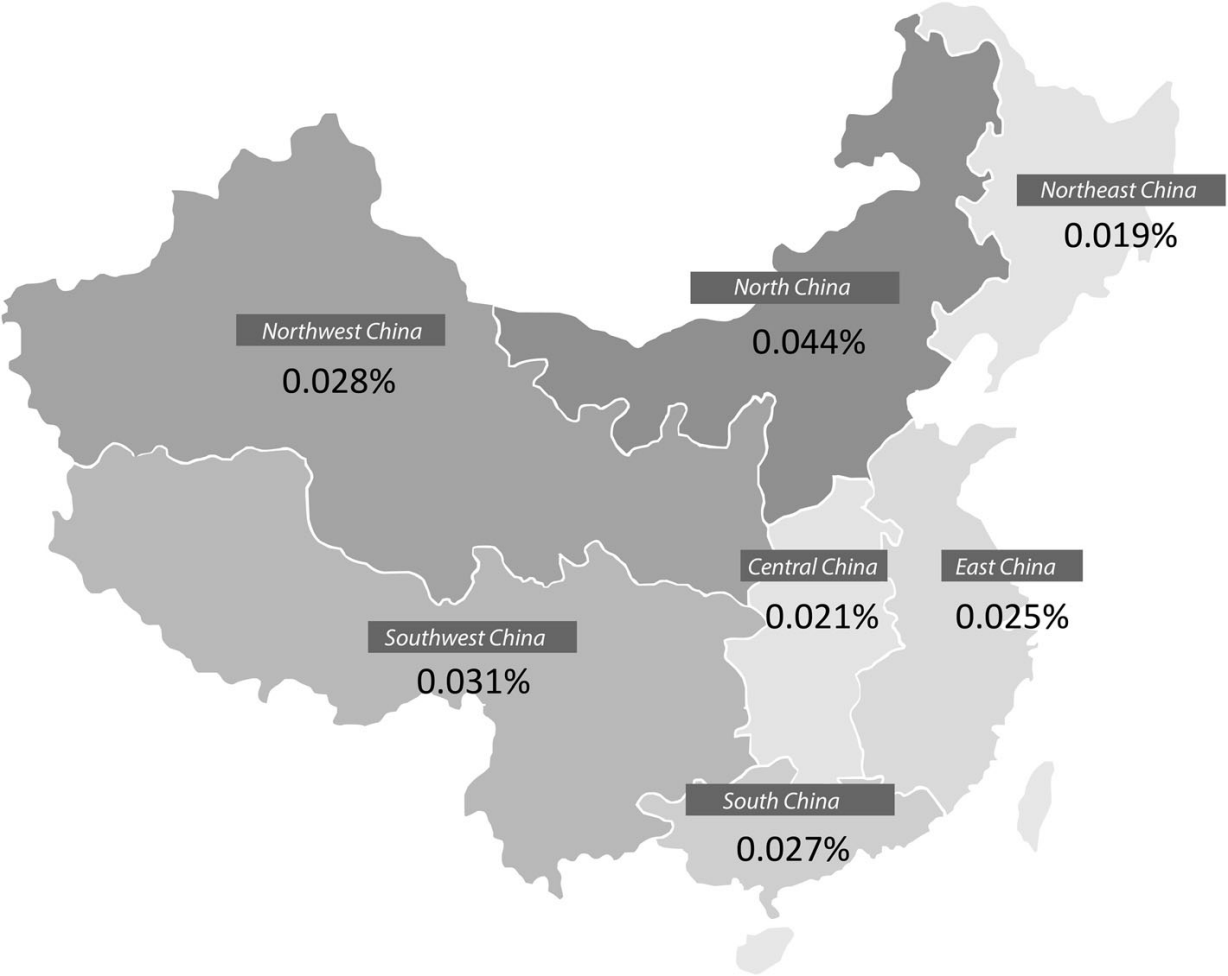
The distribution of anti-neutrophil cytoplasmic autoantibody associated vasculitis (AAV) according to Chinese geographical regions in 2015. The frequency of AAV in all inpatients in seven geographical regions, with the highest in North China (0.44‰)

There was a greater frequency of hospitalized patients with AAV in Northern China (0.44‰ of all inpatients in Northern China vs. 0.27‰ in Southern China) (Fig. 3). We analyzed the association between the frequency of AAV and the latitude of major cities in China; however, no significant association was found.

### The ethnic distribution of AAV in the study period

**Fig. 4:**
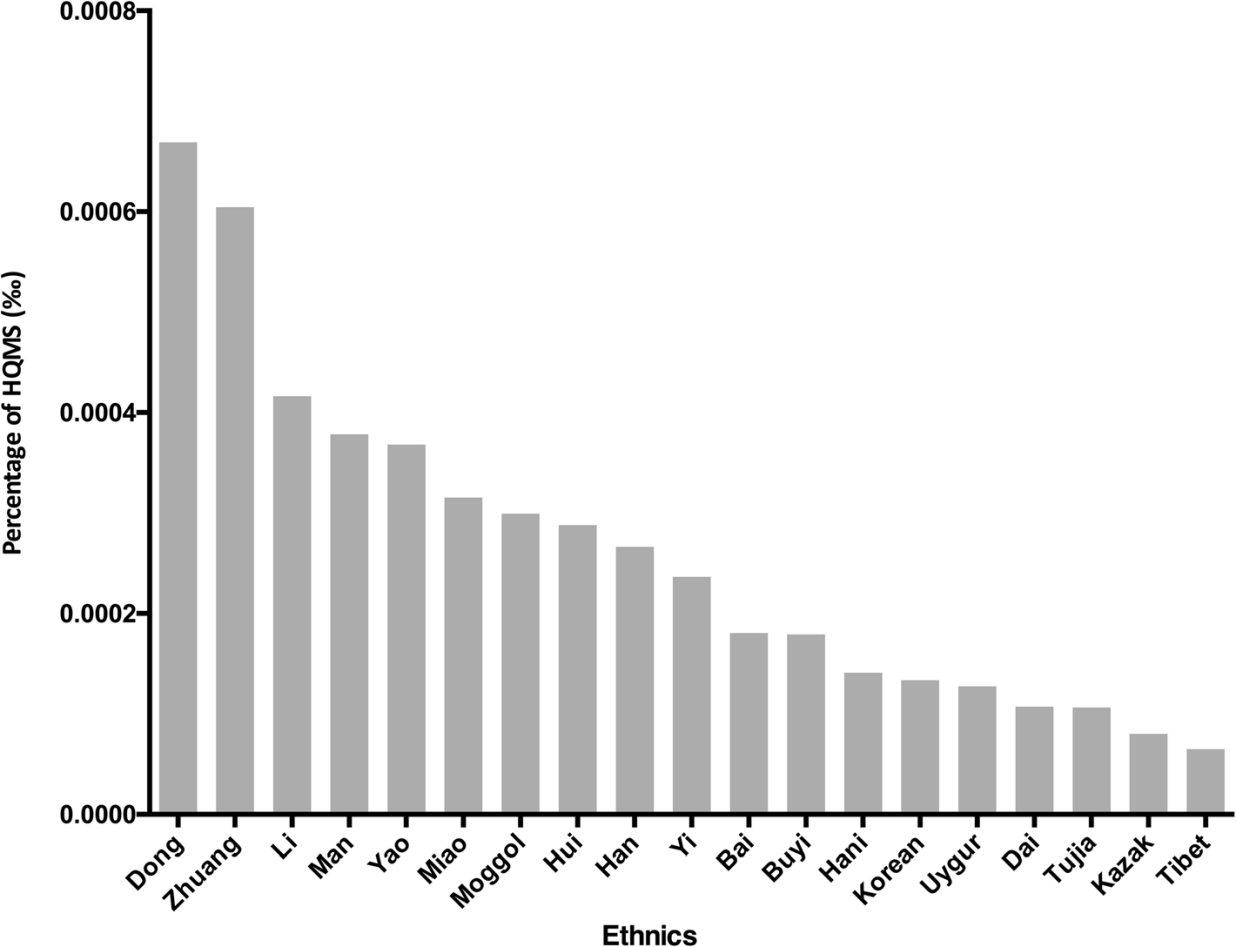
Ethnic distribution of anti-neutrophil cytoplasmic autoantibody associated vasculitis (AAV) in the Chinese population. The Dong, Zhuang and Li ethnic minorities had the highest frequencies of AAV, with a frequency of 0.67‰, 0.61‰ and 0.42‰, respectively. HQMS, Hospital Quality Monitoring System

Dong, Zhuang, and Li ethnic people had the highest frequency of AAV, with a frequency of 0.67‰, 0.61‰, and 0.42‰, respectively (Fig. 4). The Dong and Zhuang populations are mostly distributed in Southern China. However, there was no significant correlation between the proportion of ethnic groups in each province and the frequency of AAV.

### The distribution of AAV according to pollution

The pathogenesis of AAV is reported to be associated with silicon pollution [15]. We further investigated the association between various pollutants and the frequency of AAV. We analyzed the association between particular molecules and AAV using data from 946 stations covering 190 cities within 2014–2015, published by the National Air Quality Monitoring Network [28] in China, and analyzed aerosol optical depth (AOD) data from 1998 to 2014 provided by the National Aeronautics and Space Administration (NASA). Data on air sulfur dioxide, carbon dioxide, and dust were obtained from the National Bureau of Statistics. We found positive correlation between exposure to carbon monoxide and the frequency of AAV (*R*^2^ = 0.172, *P* = 0.025). However, there was no significant correlation between the frequency of AAV and air pollutents (PM 2.5, PM 10, other inhalable particulates, NO_2_, SO_2_) or water pollution. 

### The increasing frequency of AAV after the severe earthquake

**Fig. 5:**
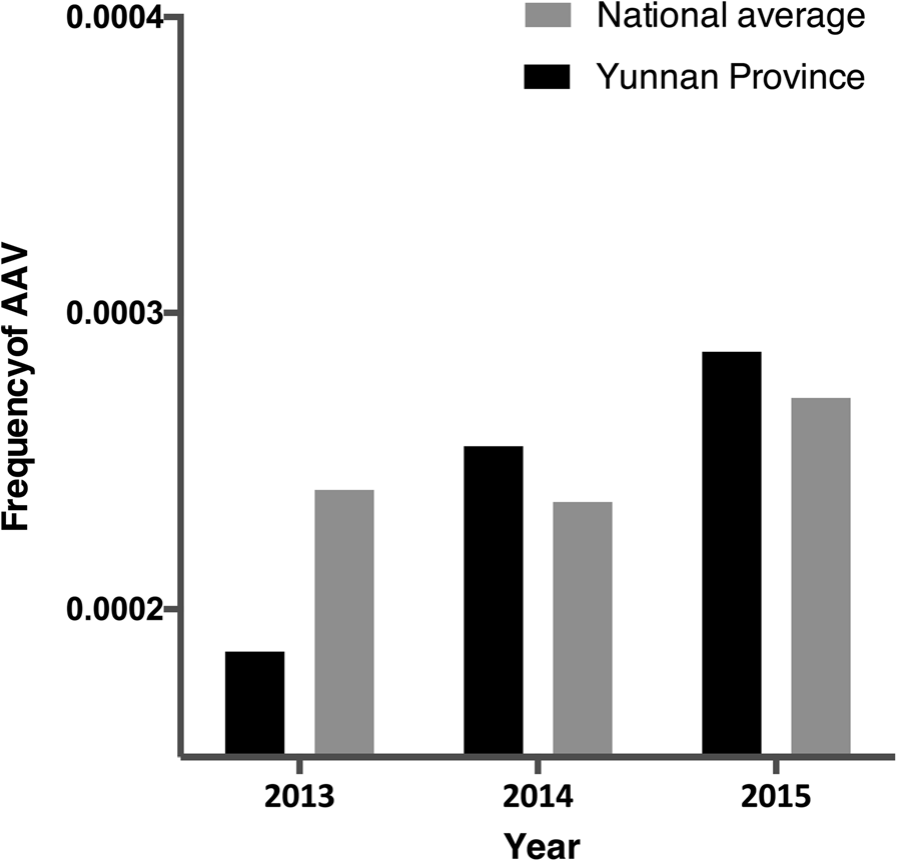
The proportion of anti-neutrophil cytoplasmic autoantibody associated vasculitis (AAV) in Yunnan province and the national average value since 2013. The frequency of AAV in Yunnan province has increased annually since 2013

On 3 Aug 2014, a major earthquake hit Yunnan province and threatened more than 1 million lives. The frequency of AAV in Yunnan province increased 1.37-fold after the earthquake, from 0.19‰ in 2013 to 0.26‰ in 2014 (Fig. 5).

## Discussion

Using a large national inpatient database covering 43,677,829 patients from 2010 to 2015, we described the epidemiological characteristics of AAV in China for the first time. We observed a changing frequency of AAV according to latitude and seasonal variations. In addition, the Dong ethnic minority had the highest proportion of patients with AAV. We also noticed that exposure to carbon monoxide (CO) might increase the frequency of AAV.

Incidence studies performed in Japan and European countries suggest that there are geographic variations in AAV [11, 29] in the northern hemisphere, while studies in Austrilia and New Zealand showed a similar trend [30, 31] (Additional file 1: Table S1). The reason is explained as genetic variation and the ultraviolet radiation gradient according to latitude [32, 33]. In the present study, the inpatients in Northern China (34.9° N to 53.4° N) had the highest proportion of patients with AAV (0.42‰), while in Central China (24.63° N to 36.37° N), Southwestern China (23.5° N to 38.4° N) and Southern China (18.2° N to 26. 4° N) the proportions were lower (0.23‰, 0.25‰, 0.28‰, respectively), which is consistent with previous reports. However, most physicians in China do not make a precise diagnosis of each subtype of AAV, i.e., GPA, MPA, or EGPA on the first page of the inpatients’ documents, probably due to the similar treatment strategy for each pathological type of AAV; thus GPA, MPA, and EGPA were not analyzed further. In addition, our data were from a hospital-based database, which can not be used to compare our data with the incidence or prevalence of AAV in other countries. However, we could still find that the disease spectrum is different between China and Japan, since the incidence of GPA in Japan is much lower than that of MPA (2.1 (0.6, 3.5) vs. 18.2 (14.3, 22.0)/million in adults, and 2.7 (− 0.8, 6.3)/million vs. 50.7 (38.3, 63.0)/million in seniors) [34], while in China it is almost the same as for MPA.

The Chinese Dong population had the highest proportion of patients with AAV in China, with incidence twofold higher than the national average. They are mainly distributed in Southwestern China and Southern China, which might also contribute to the relatively high proportion of patients with AAV compared to the provinces in the same latitude, such as Eastern China. Genome-wide association studies (GWAS) have shownd that HLA-DP (rs3117242) variants contribute to the pathogenesis of MPO-ANCA associated vasculitis, while PRTN3 (rs 62,132,295) variants might contribute to PR3-ANCA associated vasculitis [21, 34–37]. Phylogenic studies have revealed that the Chinese Dong population is a distinct population from the Han population, along with the Li and Yao populations, which also had a relatively high frequency of AAV [38]. The Dong population has been shown to have a low prevalence of type II diabetes mellitus (T2D), and seven loci were identified by GWAS to be associated with T2D in the Dong population [39]. Further genetic studies might be able to reveal the genetic variants related to AAV in this race, which might provide a promising opportunity to further explore the pathogenesis of AAV.

Earthquake and the subsequent releasing of silicon has been reported to be associated with the onset of AAV, and to exacerbate disease severity in Kobe, Japan [15, 40]. However, data from New Zealand showed an opposite result [17]. On 3 Aug 2014, there were major earthquakes in Yunnan Province in China, of which the biggest reached 6.5 on the Richter scale. According to the air pollution data published by the National Bureau of Statistics from 2010 to 2015, although there was no significant increase in PM 10, the proportion of AAV had increased 1.37-fold by 2014 in Yunnan province. This phenomenon in our present study supports the hypothesis that release of environment pollutants after earthquake might contribute to the pathogenesis of AAV. However, since our only analysis only included data from 2 years after the earthquake, we might need more follow-up data to finalize our conclusion.

In our study, we found exposure to carbon monoxide (CO) increased the frequency of AAV (*R*^2^ = 0.172, *P* = 0.025), which seems to be contradictory to the anti-inflammatory effect of CO [41]. It has been proved in animal models that CO might inhibit the activation of T cells in systematic lupus erythematosus (SLE) [42], and alleviate the inflammatory effect of peripheral mononuclear cell-derived MPO in vessels [43, 44]. However, CO could bind hemoglobin and might subsequently prevent oxygen transportation and thus result in oxygen deficiency or hypoxia in cells, which might injure the endothelium. This might suggest harmful effects of CO in vessel inflammation, which still needs in-depth exploration.

This study has several limitations. First, the data in the present study covered only 54.1% of tertiary hospitalized populations in China. Second, the diagnosis of AAV, especially GPA, MPA, and EGPA, in hospitalized patients was based on ICD-10 coding and free text from single hospitalizations with relatively low sensitivity and no laboratory data on patterns and antigenicity in patients with ANCA. Third, 77.4% of patients with AAV had not been classified according to the 2012 CHCC classification, and each subtype was not further analyzed.

## Conclusions

In conclusion, in the present study, we provided the first epidemiological data on AAV in hospitalized patients in China, which showed evident seasonal variation, geographic and ethnic clustering, and association with pollution.

### Additional files

Additional file 1: Summary of epidemiological study of AAV (per million). (DOCX 29 kb)

Additional file 2: Pollutants in major cities. (XLSX 14 kb)

Additional file 3: Pollutants in provinces. (XLSX 17 kb)

## Appendix 1

**Table 2:** The International Classification of Diseases-10 coding of AAV

Disease	ICD-10 coding
AAV	M31.802
MPA	M31.701^#^ M31.700^†^, M31.702 + G63.5^*†^
GPA	M31.301, K13.407^#^ M31.300^†^, M31.301, M31.302 + J99.1^*†^, M31.303 + N08.5^*†^
EGPA	M30.101

^#^Applicable for ICD-10 (Beijing Version 4.0) only

^†^Applicable for ICD-10 (National Standard Version1.0) only

*Abbreviations: ICD-10* International Classification of Diseases-10, *AAV* anti-neutrophil cytoplasmic autoantibodies associated vasculitis, *GPA* granulomatosis with polyangiitis, *MPA* microscopic polyangiitis, *EGPA* eosinophilic granulomatosis with polyangiitis

## Appendix 2

**Table 3:** The International Classification of Diseases-10 coding of exclusion criteria of AAV and complications

Disease	ICD-10 coding
TAK	M31.401
GCA	M31.501^#^, M31.601^#^ M31.500^†^, M31.600^†^
PAN	M30.002^#^ M30.000^†^, M30.001 + G73.7^*†^, M30.002 + G63.5^*†^, M30.003 + G63.5^*†^, M30.800^†^
KD	M30.301^#^ M30.300^†^
RA	M05.391^#^, M05.392^#^, M06.811^#^, M06.821^#^, M06.831^#^, M06.841^#^, M06.851^#^, M06.861^#^, M06.871^#^, M06.872^#^, M06.991^#^, M08.091^#^ M05.101 + J99.0^*†^, M05.102 + J99.0^*†^, M05.300+^†^, M05.301 + G63.6^*†^, M05.302 + I52.8^*†^, M05.303 + G73.7^*†^, M05.304 + I52.8^*†^, M05.305 + I32.8^*†^, M05.306 + I41.8^*†^, M05.307 + I39.8^*†^, M05.308^†^, M05.800^†^, M05.900, M06.000^†^, M06.800^†^, M06.900^†^, M06.901^†^, M06.902^†^, M06.903^†^, M06.904^†^, M06.905^†^, M06.906^†^, M06.907^†^, M06.909^†^, M08.800^†^, M08.001^†^, M08.002^†^
SLE	F06.921^#^, M32.001^#^, M32.101 + G05.8^*#^, M32.102 + I32.8^*#^, M32.104 + J99.1^*#^, M32.105 + N08.5^*#^, M32.107 + J99.1^*#^, M32.108 + G63.5^*^#, M32.109 + G99.2^*#^, M32.110 + G63.5^*#^, M32.111 + K67.8^*#^, M32.112 + N08.5^*#^,,M32.113 + N16.4^*#^, M32.901^#^ M32.000^†^, M32.100+^†^, M32.101 + N08.5^*†^, M32.102 + N16.4^*†^, M32.103 + J99.1^*†^, M32.104 + I43.8^*†^, M32.105 + I32.8^*†^, M32.106 + G63.5^*†^, M32.107 + G99.2^*†^, M32.108 + K77.8^*†^, M32.110 + G73.7^*^, M32.111 + D77^*^, M32.112 + K93.8^*^, M32.113 + H36.8^*^, M32.114 + G94.8^*†^, M32.115^†^, M32.800^†^, M32.900^†^, M32.901^†^
Sarcoidosis	D86.001^#^, D86.301^#^, D86.102^#^, D86.201^#^, D86.301^#^, D86.802 + G53.2^*#^, D86.803 + H22.1^*#^, D86.804 + M63.3^*#^, D86.805 + I41.8^*#^, D86.806 + M14.8^*#^, D86.901^#^, K13.408^#^ D86.000^†^, D86.100^†^, D86.200^†^, D86.300^†^, D86.800^†^, D86.801^†^, D86.802^†^, D86.900^†^, D86.901^†^
IBD	K50.002, K50.003^#^, K50.004^#^, K50.005^#^, K50.006^#^, K50.101, K50.102, K50.103, K50.104, K50.803^#^, K50.901^#^, K50.902 + M07.4^*#^, K51.001^#^, K51.002, K51.201^#^, K51.301, K51.901, K51.902, K51.903 + M07.5^*#^ K50.000^†^, K50.001^†^, K50.100^†^, K50.800^†^, K50.801^†^, K50.900^†^, K50.901^†^, K50.902 + M07.4^*†^, K51.000^†^, K51.001^†^, K51.003^†^, K51.004^†^, K51.005^†^, K51.200^†^, K51.201^†^, K51.202^†^, K51.203^†^, K51.300^†^, K51.302^†^, K51.303^†^, K51.400^†^, K51.401^†^, K51.800^†^, K51.900^†^, K51.903^†^, K51.904 + M07.5^*†^
LVV	I77.601^#^; I77.602^#^; I77.603^#^; I77.604^#^ M31.400^†^; M31.401^†^
HBV	54.808^#^, B18.102^#^, B18.103^#^, B18.104^#^, B18.105^#^, B18.106^#^, Z22.501^#^, Z22.502^#^, B16.001^#^, B16.002^#^, B16.101^#^, B16.103^#^, B16.104^#^, B16.105^#^, B16.201^#^, B16.202#, B16.901^#^, B16.902^#^, B16.903^#^, B16.905^#^, B18.004^#^, B18.005^#^, B18.006^#^, B18.101^#^, B18.102^#^, B18.103^#^, B18.104^#^, B18.105^#^, B18.106^#^ Z22.502^†^, Z22.503^†^, Z22.504^†^, B16.000^†^, B16.001^†^, B16.100^†^, B16.101^†^, B16.200^†^, B16.201^†^, B16.202^†^, B16.203^†^, B16.204^†^, B16.205^†^, B16.206^†^, B16.900^†^, B16.901^†^, B16.902^†^, B16.903^†^, B16.904^†^, B16.905^†^, B17.000^†^, B18.103 + N08.0^*†^, B18.104^†^, B18.105^†^, B18.106^†^, B18.107^†^, K74.602^†^
HCV	O98.402^#^, Z22.591^#^, B17.101^#^, B17.102^#^, B17.103^#^, B17.104^#^, B17.105^#^, B18.201^#^, B18.202^#^, B18.203^#^, B18.205^#^, B18.207^#^, B18.208^#^ Z22.501^†^, B18.205 + N08.0^*†^, K74.603^†^, B17.100^†^, B17.101^†^, B17.102^†^, B17.103^†^, B18.200^†^, B18.201^†^, B18.202^†^, B18.203^†^, B18.204^†^
Syphillis	A51, A52
Carcinoma	C00-D49
RPGN	N01.701^#^, N01.803^#^, N01.901^#^, N01.902^#^, N01.903^#^ N01.700^†^, N01.800^†^, N01.900^†^
Acute nephritis syndrome	N00.901^#^, N00.902^#^, N00.903^#^, N00.801^#^, N17.002^#^, N17.102^#^, N17.201^#^, N17.901^#^, N17.902^#^, N17.903^#^, N17.904^#^ N00.000^†^, N00.100^†^, N00.200^†^, N00.300^†^, N00.400^†^, N00.500^†^, N00.600^†^, N00.700^†^, N00.800^†^, N00.900^†^, N00.902^†^
NS	N04

^#^Applicable for ICD-10 (Beijing Version 4.0) only

^†^Applicable for ICD-10 (National Standard Version1.0) only

*Abbreviations: ICD-10* International Classification of Diseases-10, *TAK* Takayasu arteritis, *GCA* giant cell arteritis, PAN polyarteritis nodosa, *KD* Kawasaki disease, *LVV* large vessel vasculitis, *IBD* inflammatory bowel disease, *SLE* systemic lupus erythematosus, *RA* rheumatoid arthritis, *NS* nephrotic syndrome, *RPGN* rapidly progressive glomerulonephritis

## Abbreviations

AAV: Anti-neutrophil cytoplasmic autoantibody associated vasculitis
ANCA: Anti-neutrophil cytoplasmic autoantibody
AOD: Aerosol optical depth
CHCC: Chapel Hill Consensus Conference
CI: Confidence interval
CO: Carbon monoxide
EGPA: Eosinophilic granulomatosis with polyangiitis
GPA: Granulomatosis with polyangiitis
GWAS: Genome-wide association study
HLA: Human leukocyte antigen
HQMS: Hospital Quality Monitoring System
ICD: International Classification of Diseases
ICU: Intensive care unit
MPA: Microscopic polyangiitis
MPO: Myeloperoxidase
NASA: National Aeronautics and Space Administration
NO2: Nitrogen dioxide
PM: Particulate matter
SLE: Systemic lupus erythematosus
SO2: Sulfur oxide
