# Supplementary material for: The frequency of ANCA-associated vasculitis in a national database of hospitalized patients in China
Source: Arthritis Res Ther. 2018 Oct 4;20:226. doi: 10.1186/s13075-018-1708-7 (PMC6235226; doi:10.1186/s13075-018-1708-7)
Supplement: Supplementary file 1 — Summary of epidemiological study of AAV (per million). (DOCX 29 kb) [file 13075_2018_1708_MOESM1_ESM.docx]

**Table 1. Summary of epidemiological study of AAV (per million).**

| **Region** | **AAV** | **GPA** | **MPA** | **EGPA** |
| --- | --- | --- | --- | --- |
| 1.UK(1) | - | 10.6 | - | - |
| 2.Spain(1) | - | 18.3 | - | - |
| 3.UK(2) | 12.2 (8.0-17.7) | 5.8(2.9-9.4) | 5.0 (2.4-8.8) | 1.4 (0.3-3.9) |
| 4.Japan(2) | 14.8 (10.8-18.9) | 0 | 14.8 (10.8-18.9) | 0 |
| 5.Norfolk(UK)(3) | - | 8.5 (5.2-12.9) | - | - |
| 6.UK(4) | 144.5 | 9.7 | 8 | 2.7 |
| 7.Sweden(5) | - | 0.78 (0.74-0.82) | - | - |
| 8.Lugo(Spain)(6) | - | 4 | 11.1 | 0.88 |
| 9. Lugo(Spain)(7) | - | 2.95 | 7.91 | - |
| 10.Sweden(8) | - | 9.8 | 10.1 | - |
| 11.New Zealand(9) | 52.11 | - | - | - |
| 12.Norway(10) | - | 5.2 | - | - |
| 13.Japan(11) | 22.6 | 2.1 | 18.2 | 2.4 |
| 14.Germany(12) | 9.5~16 | - | - | - |
| 15.Germany(13) | 9.54 | 8 | - | - |

Note: Abbreviations: AAV, anti-neutrophil cytoplasmic autoantiboides associated vasculitis; GPA, granulomatosis with polyangiitis; MPA, microscopic polyangiitis; EGPA, eosinophilic granulomatosis with polyangiitis.

**Reference**

1. Watts RA, Gonzalez-Gay MA, Lane SE, Garcia-Porrua C, Bentham G, Scott DG. Geoepidemiology of systemic vasculitis: comparison of the incidence in two regions of Europe. Ann Rheum Dis. 2001;60(2):170-2.

2. Watts RA, Scott DG, Jayne DR, Ito-Ihara T, Muso E, Fujimoto S, et al. Renal vasculitis in Japan and the UK--are there differences in epidemiology and clinical phenotype? Nephrol Dial Transplant. 2008;23(12):3928-31.

3. Carruthers DM, Watts RA, Symmons DP, Scott DG. Wegener's granulomatosis--increased incidence or increased recognition? Br J Rheumatol. 1996;35(2):142-5.

4. Watts RA, Lane SE, Bentham G, Scott DG. Epidemiology of systemic vasculitis: a ten-year study in the United Kingdom. Arthritis Rheum. 2000;43(2):414-9.

5. Knight A, Ekbom A, Brandt L, Askling J. Increasing incidence of Wegener's granulomatosis in Sweden, 1975-2001. J Rheumatol. 2006;33(10):2060-3.

6. Gonzalez-Gay MA, Garcia-Porrua C. Systemic vasculitis in adults in northwestern Spain, 1988-1997. Clinical and epidemiologic aspects. Medicine (Baltimore). 1999;78(5):292-308.

7. Gonzalez-Gay MA, Garcia-Porrua C, Guerrero J, Rodriguez-Ledo P, Llorca J. The epidemiology of the primary systemic vasculitides in northwest Spain: implications of the Chapel Hill Consensus Conference definitions. Arthritis Rheum. 2003;49(3):388-93.

8. Mohammad AJ, Jacobsson LT, Westman KW, Sturfelt G, Segelmark M. Incidence and survival rates in Wegener's granulomatosis, microscopic polyangiitis, Churg-Strauss syndrome and polyarteritis nodosa. Rheumatology (Oxford). 2009;48(12):1560-5.

9. O'Donnell JL, Stevanovic VR, Frampton C, Stamp LK, Chapman PT. Wegener's granulomatosis in New Zealand: evidence for a latitude-dependent incidence gradient. Intern Med J. 2007;37(4):242-6.

10. Koldingsnes W, Nossent H. Epidemiology of Wegener's granulomatosis in northern Norway. Arthritis Rheum. 2000;43(11):2481-7.

11. Fujimoto S, Watts R A, Kobayashi S, Suzuki K, Jayne DR, Scott DG, Hashimoto H, Nunoi H. Comparison of the epidemiology of anti-neutrophil cytoplasmic antibody-associated vasculitis between Japan and the UK. *Rheumatology*, 50(10): 1916-1920, 2011.

12. Reinhold-Keller E, Herlyn K, Wagner-Bastmeyer R, Gross WL. Stable incidence of primary systemic vasculitides over five years: results from the German vasculitis register. Arthritis Rheum. 2005;53(1):93-9.

13. Reinhold-Keller E, Herlyn K, Wagner-Bastmeyer R, Gutfleisch J, Peter HH, Raspe HH, et al. No difference in the incidences of vasculitides between north and south Germany: first results of the German vasculitis register. Rheumatology (Oxford). 2002;41(5):540-9.
